# Template-switching artifacts resemble alternative polyadenylation

**DOI:** 10.1186/s12864-019-6199-7

**Published:** 2019-11-08

**Authors:** Zsolt Balázs, Dóra Tombácz, Zsolt Csabai, Norbert Moldován, Michael Snyder, Zsolt Boldogkői

**Affiliations:** 1 Department of Medical Biology, Faculty of Medicine, University of Szeged, Szeged, Hungary; 2 Department of Genetics, School of Medicine, Stanford University, Stanford, CA USA

**Keywords:** Template switching, Polyadenylation, RNA sequencing, Long-read sequencing, Direct RNA sequencing, Internal priming, cDNA sequencing

## Abstract

**Background:**

Alternative polyadenylation is commonly examined using cDNA sequencing, which is known to be affected by template-switching artifacts. However, the effects of such template-switching artifacts on alternative polyadenylation are generally disregarded, while alternative polyadenylation artifacts are attributed to internal priming.

**Results:**

Here, we analyzed both long-read cDNA sequencing and direct RNA sequencing data of two organisms, generated by different sequencing platforms. We developed a filtering algorithm which takes into consideration that template-switching can be a source of artifactual polyadenylation when filtering out spurious polyadenylation sites. The algorithm outperformed the conventional internal priming filters based on comparison to direct RNA sequencing data. We also showed that the polyadenylation artifacts arise in cDNA sequencing at consecutive stretches of as few as three adenines. There was no substantial difference between the lengths of poly(A) tails at the artifactual and the true transcriptional end sites even though it is expected that internal priming artifacts have shorter poly(A) tails than genuine polyadenylated reads.

**Conclusions:**

Our findings suggest that template switching plays an important role in the generation of spurious polyadenylation and support the need for more rigorous filtering of artifactual polyadenylation sites in cDNA data, or that alternative polyadenylation should be annotated using native RNA sequencing.

## Background

**Fig. 1:**
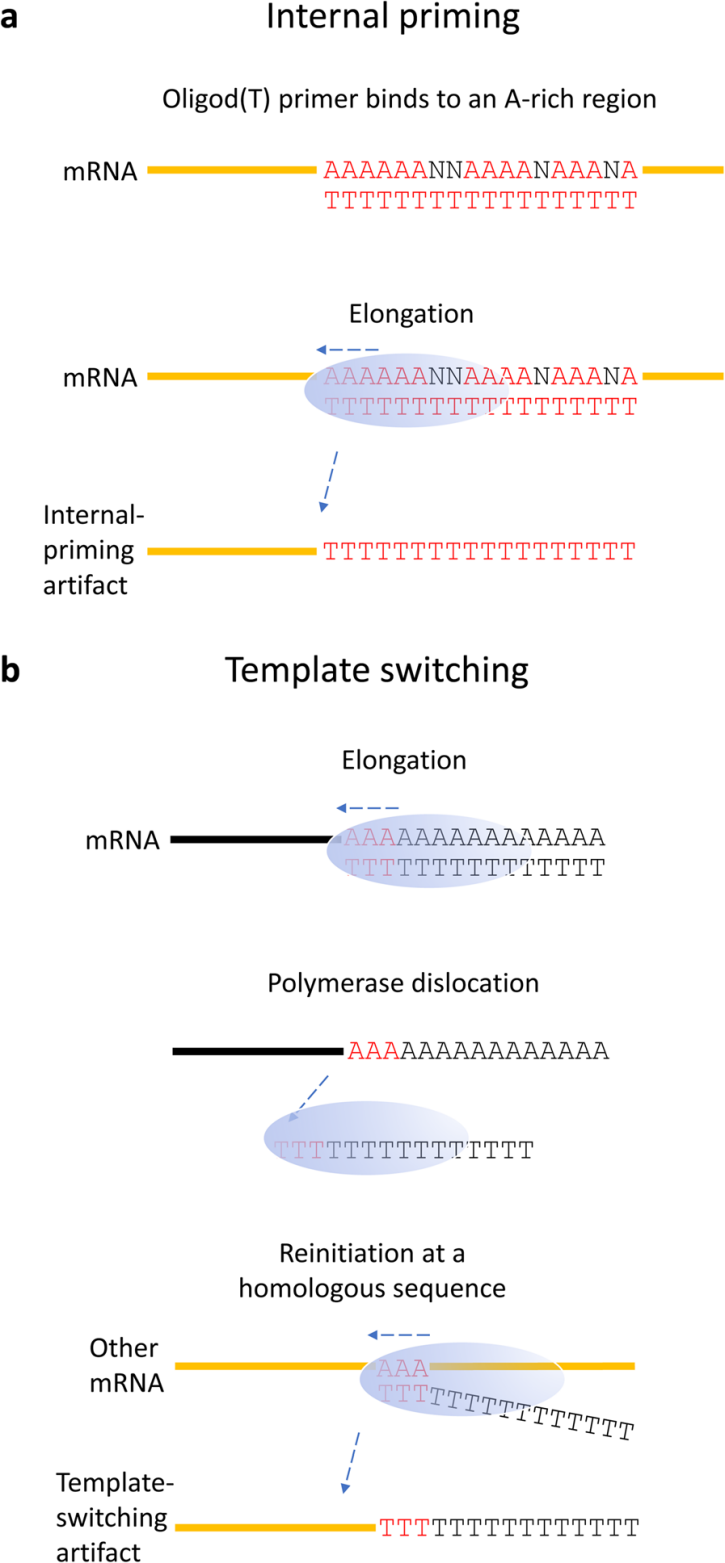
The mechanisms of internal priming and template switching. (**a**) Internal priming occurs due to the annealing of a primer to an A-rich region. A-rich regions are typically defined as genomic loci with six or more consecutive As or 12 As out of 20 nucleotides. (**b**) Template-switching artifacts are produced when the polymerase dislocates during elongation and reinitiates at a homologous sequence of another template

The majority of human genes utilize alternative polyadenylation (APA) sites [1, 2], which are a common means to increase eukaryotic coding capacity. APA is known to substantially influence gene expression [3, 4] and plays a role in disease development [5]. cDNA sequencing greatly facilitates the analysis of APA [6]; however, it is influenced by internal-priming artifacts. In the case of internal priming, the oligod(T) primer attaches to an adenine A-rich region of the transcript and initiates transcription from this region rather than the poly(A) tail [7] (Fig. 1a). RNA ligation can be applied to enable specific amplification of the 3′-ends of transcripts and to negate the effects of internal priming [8, 9]. Regular poly(A)-seq data generated using oligod(T) primers are usually filtered so that poly(A) (pA) sites in A-rich genomic regions are discarded. A-rich regions are often defined as stretches of 6 or more consecutive As or 20-nt-long windows comprising more than 60% adenines [10–14]. In a recent long-read cDNA sequencing study of the human cytomegalovirus transcriptome, we described potentially artifactual pA sites arising from homopolymer stretches—sometimes as short as only three As [15]. Based on this finding, we propose that such artifacts are produced by template switching (TS).

TS refers to the ability of DNA polymerase to discontinue elongation while still binding the newly synthesized strand and to reinitiate synthesis at a homologous locus of another nucleic acid strand [16]. (Fig. 1b). Both DNA-dependent and RNA-dependent DNA polymerases reportedly participate in TS [17, 18]. This phenomenon has been shown to occur more frequently if the concentration of the templates is high, the homologous sequences are long, or the Reverse-transcription temperature is low [19, 20]. Polymerase pausing may also facilitate TS [20]. Another study found that direct repeats of three to six nucleotides can trigger TS; however, longer homologous sequences (i.e., 12–24 nt) resulted substantially in more artifacts [21].

## Results

**Fig. 2:**
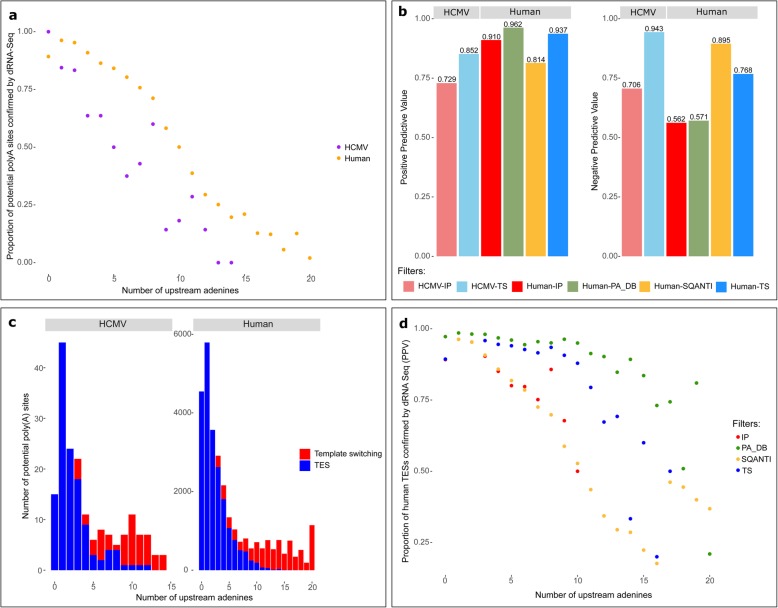
Comparison of cDNA and dRNA sequencing results of potential poly(A) sites supported by more than 10 reads. (**a**) The proportion of potential pA sites supported by dRNA sequencing for the HCMV (purple, *n* = 181) and human (orange, *n* = 30,139) datasets. (**b**) Performance of the different filtering methods. The left side shows the positive predictive value of the internal-priming (IP - red) and template-switching (TS - blue) filters based on the dRNA sequencing results (positive predictive value ~ kept sites which are also detected in dRNA Seq). Potential human pA sites were filtered using SQANTI (yellow) and also based on whether or not they occurred in PolyA_DB (green). The right side of the panel shows the proportion of potential pA sites filtered out by the different filtering options not supported by dRNA sequencing (~ negative predictive value). (**c**) Barplot of the number of potential pA sites and regions with different adenine content in the HCMV (left) and human datasets (right). The features that the filtering algorithm characterized as TES are marked in blue, whereas putative artifacts are marked in red. (**d**) The positive predictive value of the different filtering methods is shown as a function of adenine content. The HCMV results are not detailed because the low number of TESs contained in the dataset cannot provide for a meaningful analysis

We hypothesized that artifactual polyadenylation events at shorter stretches of adenines are caused by TS. To characterize artifactual and genuine transcriptional end sites, we analyzed publicly available datasets in which both direct (d) RNA and cDNA sequencing data were available for the same cell lines. Potential pA sites were determined based on the cDNA sequencing data and then compared with direct (d) RNA sequencing data to identify artifacts in the cDNA sequencing results. In total, 87,980 and 403 potential pA sites were identified in the human cDNA dataset [22] and human cytomegalovirus (HCMV) cDNA dataset, respectively [23]. Figure 2a (see also Additional file 1: Figure S1a) shows that the more As located upstream of a pA site, the less likely it was to be confirmed by dRNA sequencing. A decrease in the ratio of confirmed pA sites was already observed at relatively low numbers of As. It should be noted that not all potential pA sites missing from the dRNA sequencing data are artifacts; it is also possible that a pA site was not supported by dRNA reads simply because dRNA sequencing had lower coverage in that region. However, the adenine content of a region is not expected to reduce dRNA sequencing coverage. Therefore, decreased dRNA support for potential poly(A) sites in A-rich regions points to an increase in the number of artifacts.

Based on the hypothesis that TS—not internal priming—produces artifacts at shorter stretches of As, we devised an algorithm that differentiates between artifacts and genuine transcriptional end sites (TES). The algorithm considered the number of As in the region immediately upstream of a pA site, the number of reads in the proximity of the pA site that fell outside of A-rich regions, and the ratio of polyadenylated reads to the coverage of the region **(**Additional file 2: Figure S2). The HCMV dataset comprised of four different cDNA sequencing experiments. The potential pA sites called differently in different experiments (e.g. called as artifact according to the Sequel data but called as TES in the MinION sequencing data) were regarded as TESs. Of the 859 calls, 24 were discordant out of which 18 were confirmed by dRNA sequencing to be genuine TESs, which suggests that the algorithm is more likely to generate false negative than false positive results. The algorithm proved to have both a higher positive predictive value (75.1% instead of 72.2% in the human and 67.4% instead of 50.4% in the HCMV samples) and a higher negative predictive value (88.0% instead of 79.7% in the human and 93.0% instead of 72.7% in the HCMV samples) than the conventional internal priming filtering method (≥6 consecutive or ≥ 12 As in a 20-nt region) (Additional file 1: Figure S1b). The positive predictive value was increased by excluding from the analysis all potential pA sites with 10 or fewer poly(A) + reads in the 21-nt window around them (from 75.2 to 93.7% in the human and from 67.4 to 85.2% in the HCMV samples) while the negative predictive value did not change so markedly (from 88.0 to 85.2% and from 93.0 to 76.7%). It should be emphasized that the accuracy estimates are based on the dRNA sequencing because the ground truth is unknown. Therefore, the actual values are expected to be different, however the trends in the relations and changes in these values in consequence of the different filtering methods are expected to be similar. The generally lower estimates for positive predictive value, but higher negative predictive values in the HCMV sample compared to the human sample are most likely to be the result of lower dRNA/cDNA coverage ratios in the HCMV sample. These differences, however, do not influence the comparison of the filtering methods. Excluding such sites further reduced the number of putative artifacts (from 142 to 53 in the HCMV dataset and 40,840 to 8366 in the human dataset) relative to the number of putative TESs (from 261 to 128 in the HCMV dataset and 47,140 to 21,773 in the human dataset). Even when only high-confidence sites (supported by > 10 reads) were considered, the algorithm performed better than the internal priming filtering method (Fig. 2b). Most potential pA sites contained few adenines, whereas the majority of putative artifacts occurred in regions with a high adenine content (Fig. 2c and Additional file 1: Figure S1c**)**. Nevertheless, many putative artifacts were detected in regions with as few as 3–5 As, and the likelihood of these artifactual pA sites to be detected by dRNA sequencing did not decrease when these sites contained more As in a 20-nt window (Additional file 3: Table S1). The list of potential human pA sites was also compared with PolyA_DB, a database of poly(A) sites validated by the 3’READS+ method, which uses RNase H digestion and RNA ligation to prevent internal priming [24]. The sites confirmed by PolyA_DB data were the most likely to have been confirmed by dRNA sequencing data, although many sites not in PolyA_DB were also detected by dRNA sequencing. Interestingly, potential pA sites in PolyA_DB were less likely to have been confirmed by dRNA sequencing if they were in A-rich regions, although this phenomenon was not as prominent as that for other pA sites (Fig. 2d and Additional file 1: Figure S1d). While filtering based on presence in the PolyA_DB led to the highest positive predictive values (86.7% for all sites and 96.2% for sites with at least 10 confirming reads), this filtering also discards many genuine poly(A) sites (reflected by a negative predictive value of 78.0% for all sites and 57.1% for sites with at least 10 confirming reads). The poor negative predictive values are due to the fact that PolyA_DB is based on data from only a small variety of tissues and different experimental conditions are expected to result in poly(A) sites that are not found in the database. The quality control pipeline SQANTI [25] also offers to filter internal priming artifacts. The pipeline requires a transcript annotation and – at defaults settings – its internal-priming filter only filters out novel pA sites that have at least 17 As in the upstream 20 nucleotides. None of the putative HCMV pA sites had so many upstream As, therefore this filter of the SQANTI pipeline would not flag any of the potential pA sites as artifacts. In the human dataset, SQANTI achieved the highest negative predictive values, but also the lowest positive predictive values (Fig. 2b and Additional file 1: Figure S1b). Owing to the fact that the SQANTI pipeline only filtered pA sites in extremely A-rich regions, almost all of the discarded sites were shown to be artifacts, however many artifactual sites were not filtered out. All filtering methods performed worse at regions containing more than ten adenines (Fig. 2d and Additional file 1: Figure S1d).

**Fig. 3:**
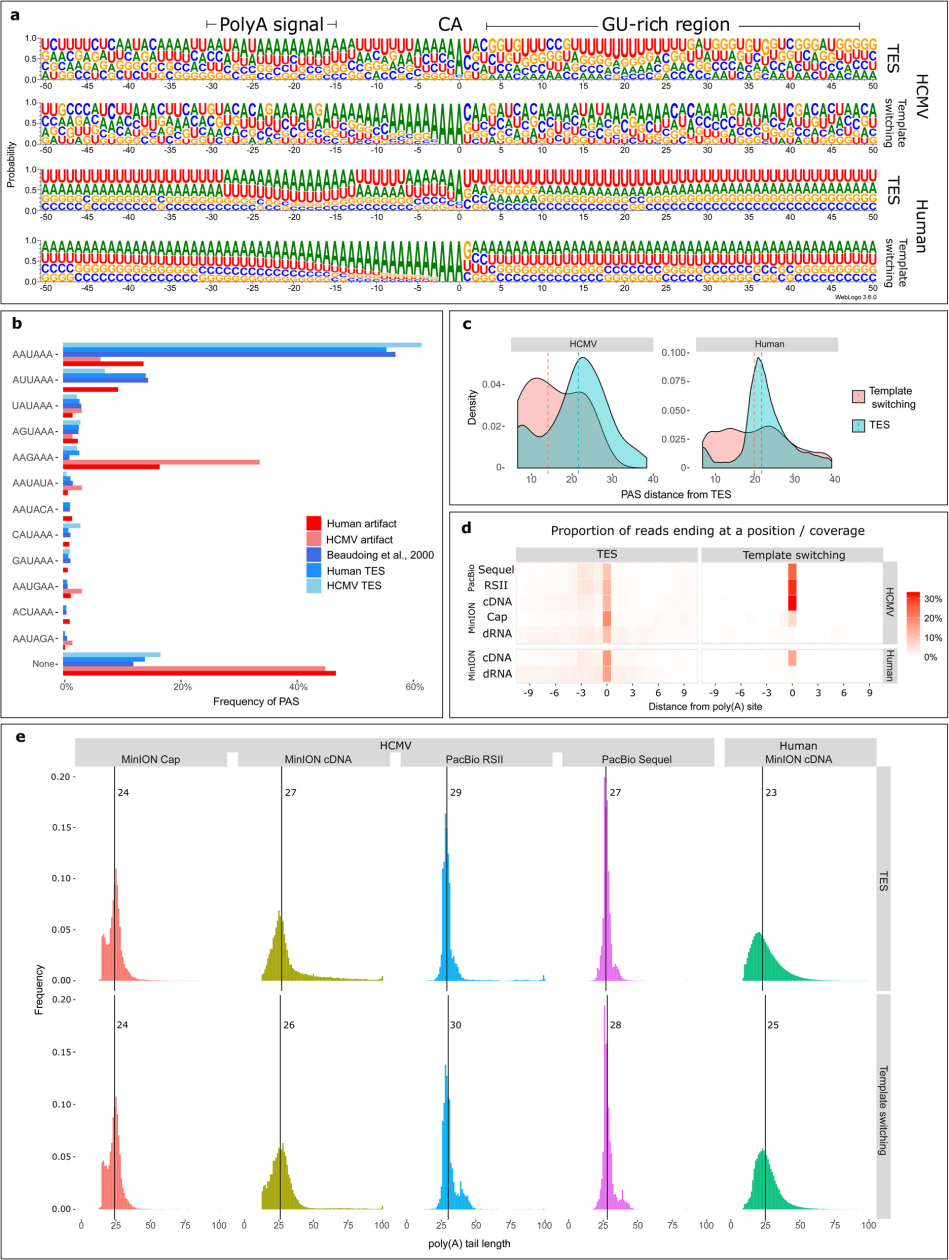
Putative template-switching artifacts differ from putative transcriptional end sites. (**a**) The nucleotide composition of the regions surrounding (±50 nt) putative TESs and putative template-switching artifacts in the HCMV dataset (above) and the human dataset (below). Common polyadenylation motifs are marked on the top of the panel. Zero denotes the location of potential pA sites. (**b**) Polyadenylation signals detected upstream of TESs (blue) and putative artifactual pA sites (red). Data for human PAS usage taken from reference [26] are shown in purple. (**c**) Density plot of the distance between the detected PASs and potential pA sites at positions characterized as TESs (blue) and at positions characterized as artifactual sites (red). (**d**) Heatmap showing the proportion of reads ending at a given nucleotide in the vicinity (±10 nt) of a potential pA site. The values of all high-confidence (supported by > 10 reads) potential pA sites are averaged. Darker colors mean that a higher proportion of alignments ended at a given position. The separate cDNA sequencing experiments from the HCMV dataset are shown separately. (**e**) Poly(A) tail length distributions measured by cDNA at TES (above) and at artifactual sites (below). The medians are shown as vertical lines. Apart from the median values which may be somewhat dislocated by to A-rich regions, it is important to note that long poly(A) tails (> 40 nucleotides) are just as prevalent in the genuine and in the artifactual groups

The putative TESs identified by the algorithm differed greatly from the putative TS artifacts (Fig. 3). The nucleotide composition surrounding TESs showed specific motifs commonly observed around cleavage sites (Fig. 3a). Putative TESs were often preceded by common polyadenylation signals (PAS), whereas putative TS artifacts generally lacked such signals (Fig. 3b). PAS usage in HCMV, like in other herpesviruses [27], is very similar to its host. Accordingly, the PAS usage of HCMV TESs was very similar to that of human TESs, but different from putative artifacts (Fig. 3b). In cases where putative artifacts were preceded by PASs, the signal was often not at the expected distance of 25 nt, as observed at putative TESs (Fig. 3c). Polyadenylation at a given pA site does not always occur at the same nucleotide; rather, it may occur at any of several nucleotides around the most frequently cleaved nucleotide [15, 26, 28]. This phenomenon was observed at putative TESs in both the human and HCMV datasets but absent at artifactual pA sites (Fig. 3d). The accumulation of many artifactual reads at certain positions is due to an erroneous alignment to homopolymer As, whereas the genuine cleavage sites are more spread around a given position. Figure 3d also shows that while different HCMV cDNA sequencing experiments often confirmed the same artifactual sites, dRNA sequencing generally did not confirm the sites that were called artifactual by the algorithm. The anchored oligod(T) primers used for reverse transcription in all experiments were 20-nt long. While anchors increase the probability of the oligonucleotides priming at the very start of the poly(A) tail, longer poly(A) tails were observed in many cases, which may be due to annealing of the anchored primer to the downstream part of the poly(A) tail. However, if artifactual pA sites were produced by annealing of the oligod(T) primers, the expected length of the poly(A) tail at these loci should be close to 20 nt with some deviation caused by polymerase and sequencing errors. Notwithstanding, the lengths of poly(A) tails sequenced at spurious pA sites did not differ from those measured at real cleavage sites (Fig. 3e).

## Discussion

We analyzed poly(A)^+^ cDNA sequencing data of two species (human and HCMV), stemming from three different long-read sequencing platforms (RSII, Sequel and MinION), generated by three different library preparation methods (Iso-Seq, Cap and poly(A)-selection, as well as only poly(A)-selection), and then compared them to dRNA sequencing data obtained by the MinION platform. Our analyses confirmed that artifacts arising in A-rich regions complicate the study of alternative polyadenylation. This phenomenon is generally accredited to internal priming [7]. Given our findings, we argue that TS is more likely to be responsible for these artifacts as many artifacts were detected in regions with rather few As (sometimes three to five), which make oligod (T) primer binding unlikely. Further, it would be expected that reads ending at artifactual sites produced by internal priming would not contain poly(A) tails substantially longer than the oligod (T) primer. However, we found that poly(A) tails at artifactual sites were longer than the primer and not shorter than poly(A) tails at bona fide TESs. We thus developed a filtering algorithm to differentiate TS artifacts from genuine TESs. Based on comparison with dRNA sequencing data, the filtering algorithm performed better than conventional internal priming filters. We suggest that, although internal priming is likely to contribute to the number of artifacts in very A-rich regions, artifacts in regions with lower adenine content are generated by TS. The positive predictive value of the template-switching filter was superseded by the filtering based on presence in the PolyA_DB, however the negative predictive value of that filter was low. The SQANTI algorithm, on the other hand, was less stringent on filtering out artefacts, but the discarded sites were more likely artefactual. Our filtering algorithm provides more balanced accuracy measures without a need for an existing transcript annotation, nor a curated pA site database.

Even though it was not part of the filtering criteria, sites that the algorithm classified as TESs were likely to contain consensus polyadenylation motifs. This result indicates that machine learning algorithms can distinguish even more specifically between TESs and artifacts using more sequence information. However, a large training dataset would be required for machine learning to be efficient, and such datasets are not available at the time.

We have shown that the TS is prevalent in both the viral and the human dataset. Nevertheless, large differences were observed in the proportion of detected artefactual and genuine poly(A) sites. These could potentially be attributed to the fact that the human genome has a lower GC-content (40.9%) than the HCMV (57.2%), therefore there are more A-rich regions in the human genome. However, another explanation is that the number of genuine polyadenylation sites is finite. Once all the genuine polyadenylation sites have been detected, any increase in coverage can only increase the number of false positives. In the small viral genome, it is more feasible to capture all the genuine sites than in the large human genome. The large overall coverage of cDNA reads, especially the high cDNA to dRNA ratio in the HCMV dataset are likely to have contributed to the lower positive and higher negative predictive values in the HMCV samples.

Our findings were obtained using long-read sequencing datasets. While it may seem sensible to extend our conclusions to short-read sequencing data and other results obtained by cDNA sequencing, it must be noted that some aspects of long-read sequencing promote the production of template-switching artifacts. Firstly, long-read sequencing usually necessitates reverse transcription of the whole transcript, not only its most 3′ fragment, which is an option for short-read sequencing. Reverse-transcribing more genomic regions provides more potential templates for TS. Secondly, SMART technology, which is widely used in long-read sequencing studies to produce full-length transcripts, requires ideal conditions for TS [29, 30]. Whereas the SMART protocol allows reverse transcription to be carried out at 50 °C, the second strand synthesis in the same reaction mixture must occur at 42 °C to allow strand switching. The characteristics of long-read sequencing library preparation increase the impact of TS; nevertheless, similar artifacts could influence other reverse-transcription-based methods as well.

## Conclusions

TS is known to produce cDNA artifacts, however its effects on the analysis alternative polyadenylation have never been discussed until now. Considering that the poly(A) tail is likely the most frequent template in most transcriptomic libraries, polyadenylation artifacts may be even more prevalent than the more reviewed splicing artifacts. The effects of TS on short-read sequencing can be mitigated by higher reverse-transcription temperatures [31] or by employing high read-count thresholds that are easier to implement due to the higher throughput of these sequencing methods. Long-read cDNA sequencing approaches are currently more prone to TS artifacts, but these artifacts can be ruled out by dRNA sequencing or curated pA-site databases when available for the studied organism. If such datasets are unavailable or inappropriate, we advise strict filtering that also considers the effects of TS. The filtering method presented here can be applied to data from any long-read sequencing platform and performs better than the conventional filtering method. An important advantage of this filtering method is its higher sensitivity that allows utilization of more data, which is crucial for long-read RNA sequencing as it has a lower throughput than short-read sequencing methods [32].

## Methods

### Data acquisition

Two long-read cDNA and dRNA sequencing datasets were downloaded and analyzed during the study. (1) The human cDNA and dRNA sequencing FASTQ reads of the Nanopore WGS Consortium (https://github.com/nanopore-wgs-consortium/NA12878/blob/master/RNA.md) were generated by extracting RNA from the GM12878 human cell line (Ceph/Utah pedigree) and sequenced on MinION flow cells (FLO-MIN106) using R9.4 chemistry (SQK-RNA001 and SQK-LSK108 kits) [22]. This dataset will be referred to as the human dataset. (2) Previously published [23, 33] data of the lytic HCMV transcriptome were downloaded from the European Nucleotide Archive, from the accession numbers PRJEB22072 (https://www.ebi.ac.uk/ena/data/view/PRJEB22072) and PRJEB25680 (https://www.ebi.ac.uk/ena/data/view/PRJEB25680). RNA was isolated from HCMV-infected (strain Towne, ATCC VR-977) human embryonic fibroblast cells (MRC-5, ATCC CCL-171) and sequenced on the RSII and Sequel platforms of Pacific Biosciences using the Iso-Seq library preparation protocol and on the MinION platform using the SQK-RNA001 and SQK-LSK108 kits and another cDNA library was prepared combining the SQK-LSK108 and the TeloPrime Full-Length cDNA Amplification Kit (Lexogen) to select for capped RNA molecules. In this latter experiment, the TeloPrime kits own enzymes were used for poly(A)-selection. This dataset containing results from five sequencing libraries (RSII and Sequel Iso-Seq libraries, MinION cDNA, cap-selected MinION cDNA and MinION dRNA libraries) is referred to in the text as the HCMV dataset.

### Mapping and read processing

The computational pipeline of the study is summarized in Additional file 2: Figure S2. The processing steps for the human and HCMV data were the same. The reads were mapped using minimap2 [34] to the human genome (hg19) and to the HCMV strain Towne varS genome (LT907985). Reads from the HCMV dataset were only mapped to the viral genome, reads from the viral infected host were not used. The mapper settings were “-ax splice -Y -C5” for the cDNA and “-ax splice -uf -k14” for the dRNA sequencing reads. Coverage and dRNA read endings were determined using bedtools [35]. As dRNA sequencing does not accurately sequence the terminal poly(A) tail of the reads, every dRNA read ending was counted. A genomic locus was confirmed as a poly(A) site confirmed by dRNA sequencing, if at least 0.5% of the overlapping dRNA reads ended in the 21-nt window (10 nt upstream + the locus + 10 nt downstream = 21 nt) around the locus.

### Identifying potential poly(a) sites in the cDNA sequencing data

The LoRTIA toolkit (https://github.com/zsolt-balazs/LoRTIA) was used to identify potential poly(A) sites in the cDNA sequencing data. A genomic locus was considered a potential poly(A) site when at least two reads and at least 0.1% of the overlapping reads ended at a given nucleotide. In a 21-nt window, the genomic position with the highest number of poly(A) + reads was selected as the potential poly(A) site. The separate experiments of the HCMV dataset were analyzed separately and the results were joined to create the list of potential HCMV poly(A) sites. The LoRTIA toolkit was also used to mark reads which ended in A-rich genomic regions (three or more consecutive As as potentially artefactual reads). When characterizing high confidence calls only the sites where more than ten reads ended in a 21-nt window around the locus were analyzed.

### Defining A-rich regions

We have deployed a slightly different definition of A-rich regions than it is commonly used in the literature. A common approach is to count the number of consecutive As in a region surrounding the poly(A) site. Another method is to count the number As in a given, often 20-nt-long window (because 20 nt is the primer length). Instead, we iterated the 20 nt upstream of a potential poly(A) site and incremented a counter each time an A was iterated, all the other nucleotides were counted as − 1. If the counter reached − 1, the iteration was halted and the highest count was regarded as the A-count of the region (Additional file 2: Figure S2). This method of defining A-rich regions combines the strengths of the previously described methods. It (1) gives more weight to As close to the polyA-site, which are more likely to contribute to the generation of artefacts. However, (2) it still considers the broader environment of the site, not just consecutive stretches of As.

### Filtering out template-switching (TS) artefacts

The potential poly(A) sites which were not at A-rich loci, were accepted by our script as transcriptional end sites. The potential poly(A) sites at A-rich loci were accepted as TES if the number of reads in a 21-nt window around that loci contained either more reads which ended in a non-A-rich region than reads which ended in an A-rich region or a proportion of overlapping reads greater than $\frac{0.8}{1+{2}^{-100\left(\frac{1}{20-n}-0.08\right)}},$ where *n* is the number of As in the A-rich region. The potential poly(A) sites which did not meet these requirements were classified as TS artefacts. The genomic loci which contained at least 100 times more 5′-ends from the opposite strand than 3′-ends were also classified as artefactual poly(A) sites. The sites which were classified as TS artefacts, but were no further than 50 nt away from sites that were classified as TES, were excluded from comparisons and sequence analyses in order to prevent the characteristics of the TES to interfere with the characteristics of TS artefacts (e.g. a polyadenylation signal that is upstream of a TES could also be upstream of a TS artefact, similarly, a direct RNA read ending in the proximity of a TES could also fall into the 21-nt window of an artefactual site).

### Detecting polyadenylation signals

The 12 most commonly utilized human PAS [26] were searched for in the region 40 nt upstream of each potential poly(A) site. Only exact matches were accepted. If the sequence upstream of a potential poly(A) site contained multiple PAS sequences, the most common PAS was assigned to the site.

### Measuring poly(a) tail length

The length of the sequenced poly(A) tail of the cDNA reads was measured for each read that contained a poly(A) tail recognized by the LoRTIA toolkit. The sequence containing the 3′-terminal 30 nt of the mapped part of the read and the first 150 nt of the 3′-terminal soft clipped (unmapped) part of the read was aligned to a stretch of 180 As using the Smith-Waterman algorithm (match score: + 2, mismatch score: − 3, gap opening score: − 3, gap extension score: − 3). The number of As in the best local alignment was counted as the length of the poly(A) tail. Measuring the poly(A) tail length this way may overestimate poly(A) tail length in A-rich regions, however it allows for a more accurate measurement in error prone reads.

### Comparison with the PolyA_DB

The PolyA_DB data [36] (Release 3.2) containing polya(A) sites which were identified using the 3’READS+ method were downloaded from the projects webpage (http://exon.umdnj.edu/polya_db/v3/misc/download.php). Potential poly(A) sites were considered confirmed by the PolyA_DB dataset if the database contained poly(A) sites in the 21-nt window of the potential poly(A) sites.

### Filtering using SQANTI

The potential pA sites in the human sample were filtered using SQANTI [25], a quality control software frequently used in long-read transcriptomics. The tool was run with the default settings of filtering out pA sites with higher than 80% A-content in the upstream 20 nucleotides.

## Supplementary information

**Additional file 1:** **Figure S1.** Comparison of the cDNA and dRNA sequencing results of all potential poly(A) sites. In addition to Fig. 2, Additional file 1 Figure S1 contains the results of potential poly(A) sites that were supported by 10 or less cDNA sequencing reads as well. (a) The proportion of potential poly(A) sites that were supported by dRNA sequencing for the HCMV (purple, *n* = 403) and the human (orange, *n* = 87,980) datasets. (b) The performance of the different filtering methods. The left side shows the true positive predictive value of the internal-priming (red) and template-switching (blue) filters based on the dRNA sequencing results (positive predictive value ~ kept sites which are also detected in dRNA Seq). The human potential poly(A) sites were filtered using SQANTI (yellow) and also based on whether or not they were found in the PolyA_DB (green). The right side of the panel shows the proportion of potential poly(A) sites filtered out by the different filtering options, which were not supported by dRNA sequencing (~ positive predictive value). (c) Barplot of the number of potential poly(A) sites in regions with different adenine-content in the HCMV (left) and (right) human dataset. The features that the filtering algorithm characterized as TES are marked in blue, the putative artefacts are marked in red. (d) The positive predictive value of the different filtering methods is shown as a function of the adenine content. The HCMV results are not detailed because the low number of TESs contained in that dataset would not provide for a meaningful analysis.

**Additional file 2:** **Figure S2.** Overview of the analysis. (a) A summary of the pipeline. Specific steps are explained in detail in boxes. (b) Identifying potential poly(A) sites. The criteria used by the LoRTIA toolkit to detect poly(A) sites are listed. The number of polyadenylated reads ending at a given nucleotide had to be at least two and at least 0.1% of the overlapping reads for the site to be considered a potential poly(A) site. If multiple sites fulfilled these criteria in a 21-nt window, the site with the highest number of supporting reads was accepted. (c) The algorithm for determining the adenine content of a region. The counting started from the poly(A) site, and each A increased, while every different letter decreased the counter by one. The counting continued until maximum 20 nucleotides or until the counter reached − 1. The highest value of the counter was regarded as the adenine content. (d) Criteria for characterizing a potential poly(A) site as a TES. One criterion was that the number of reads ending in not A-rich regions (less than three As) in a 21-nt window, had to be higher than the number of reads ending in A-rich regions (three or more As). The second criterion was that the number of supporting reads had to be higher than the percentage of overlapping reads calculated by a logistic function that takes into consideration the adenine content of the region. A potential poly(A) site was classified as a TES if any of the two criteria was fulfilled.

**Additional file 3:** **Table S1.** Negative predictive value in A-rich regions in the human dataset. The percentage of putative TS artifacts not supported by the dRNA sequencing experiment (i.e. negative predictive value) is presented in A-rich regions of different adenine content calculated by two algorithms. If artifacts were mainly caused by internal priming, it would be expected that true negative calls accumulated in regions which have a high number of adenines despite having few consecutive adenines close to the artifactual pA site. The cells are colored according to their negative predictive value.

## Data Availability

The human dataset used in this study was downloaded from GitHub site of the WGS consortium (https://github.com/nanopore-wgs-consortium/NA12878/blob/master/RNA.md). HCMV data were downloaded from the European Nucleotide Archive from accession numbers PRJEB22072 (https://www.ebi.ac.uk/ena/data/view/PRJEB22072) and PRJEB25680 (https://www.ebi.ac.uk/ena/data/view/PRJEB25680). The custom scripts used for the analysis have been made available (https://github.com/zsolt-balazs/template-switching_filter).

## Abbreviations

APA: Alternative polyadenylation
HCMV: Human cytomegalovirus
pA site: polyadenylation site
PAS: Polyadenylation signal
TES: Transcriptional end site
TS: Template switching
