# Supplementary figures and images for: Template-switching artifacts resemble alternative polyadenylation
Source: BMC Genomics. 2019 Nov 8;20:824. doi: 10.1186/s12864-019-6199-7 (PMC6839120; doi:10.1186/s12864-019-6199-7)

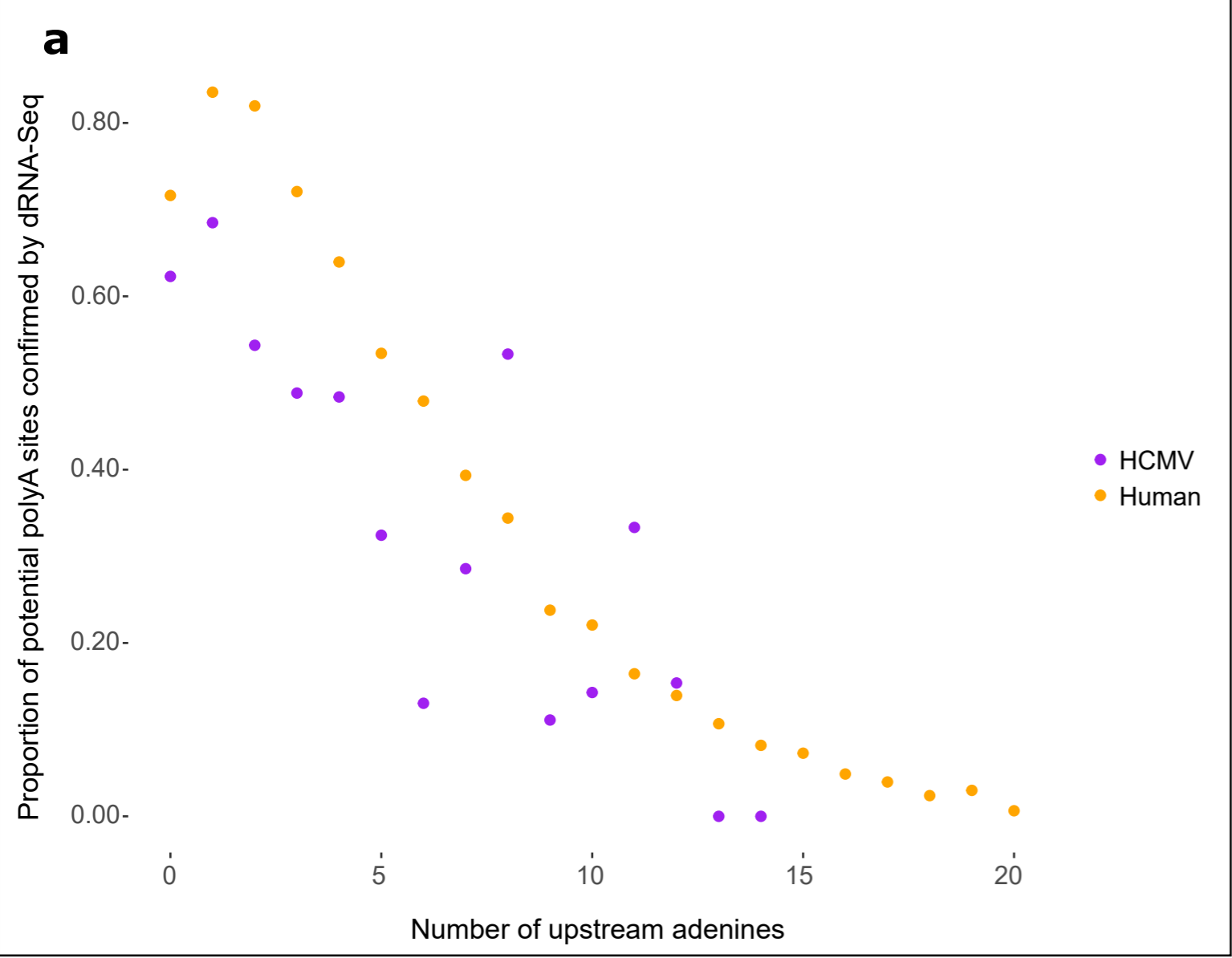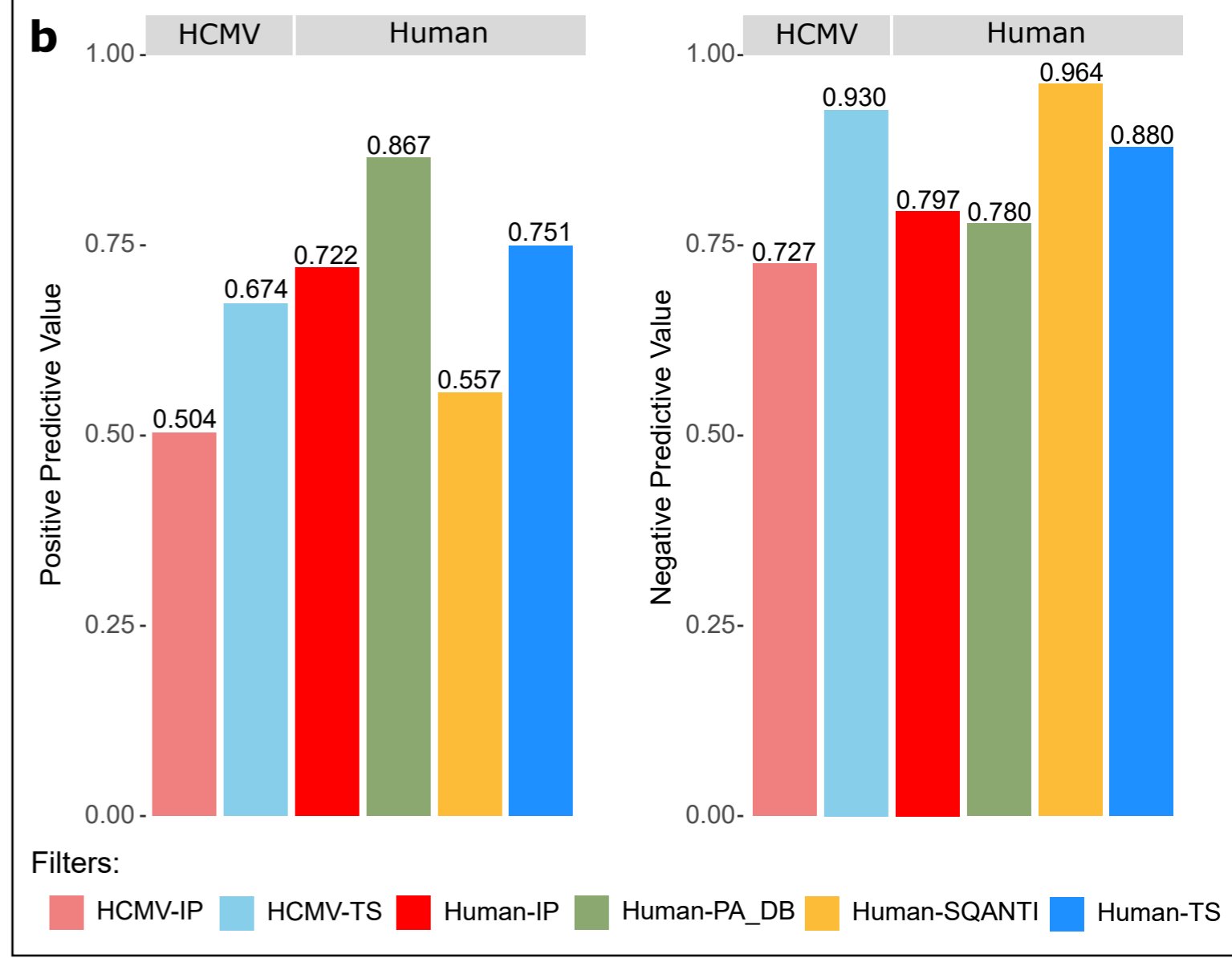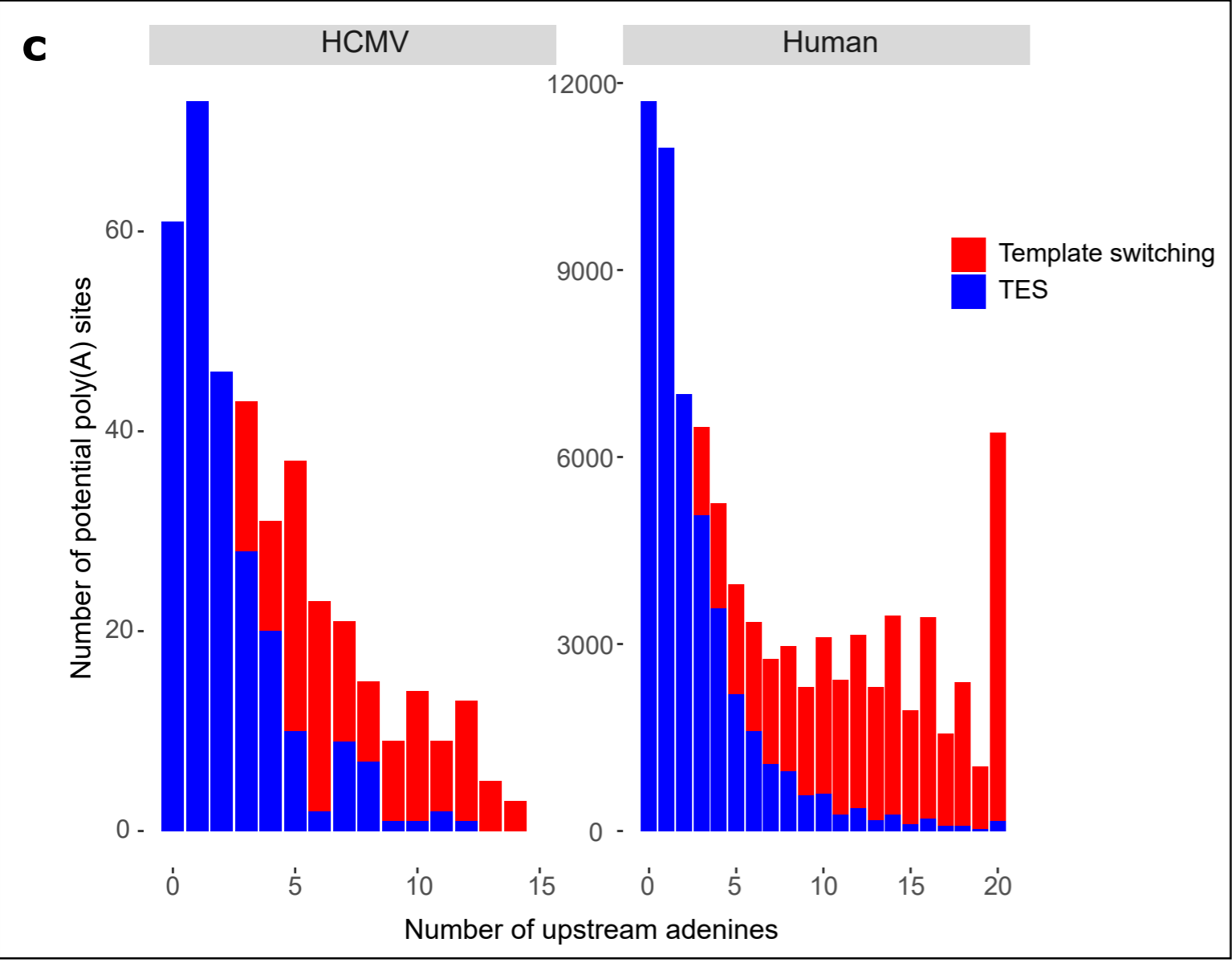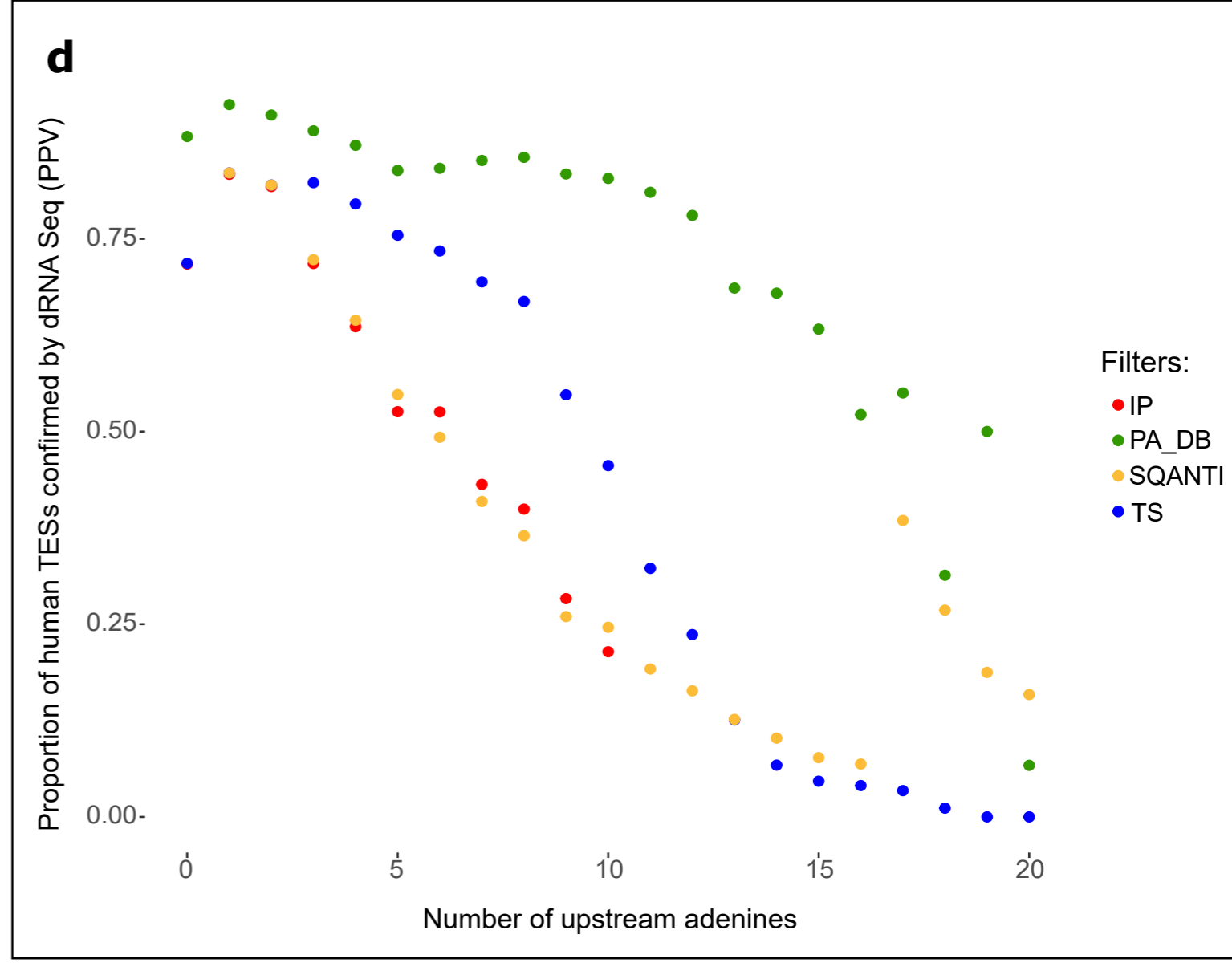

Supplement: Supplementary file 1 — Additional file 1: Figure S1. Comparison of the cDNA and dRNA sequencing results of all potential poly(A) sites. In addition to Fig. 2, Additional file 1 Figure S1 contains the results of potential poly(A) sites that were supported by 10 or less cDNA sequencing reads as well. (a) The proportion of potential poly(A) sites that were supported by dRNA sequencing for the HCMV (purple, n = 403) and the human (orange, n = 87,980) datasets. (b) The performance of the different filtering methods. The left side shows the true positive predictive value of the internal-priming (red) and template-switching (blue) filters based on the dRNA sequencing results (positive predictive value ~ kept sites which are also detected in dRNA Seq). The human potential poly(A) sites were filtered using SQANTI (yellow) and also based on whether or not they were found in the PolyA_DB (green). The right side of the panel shows the proportion of potential poly(A) sites filtered out by the different filtering options, which were not supported by dRNA sequencing (~ positive predictive value). (c) Barplot of the number of potential poly(A) sites in regions with different adenine-content in the HCMV (left) and (right) human dataset. The features that the filtering algorithm characterized as TES are marked in blue, the putative artefacts are marked in red. (d) The positive predictive value of the different filtering methods is shown as a function of the adenine content. The HCMV results are not detailed because the low number of TESs contained in that dataset would not provide for a meaningful analysis. [file 12864_2019_6199_MOESM1_ESM.pdf]

**a**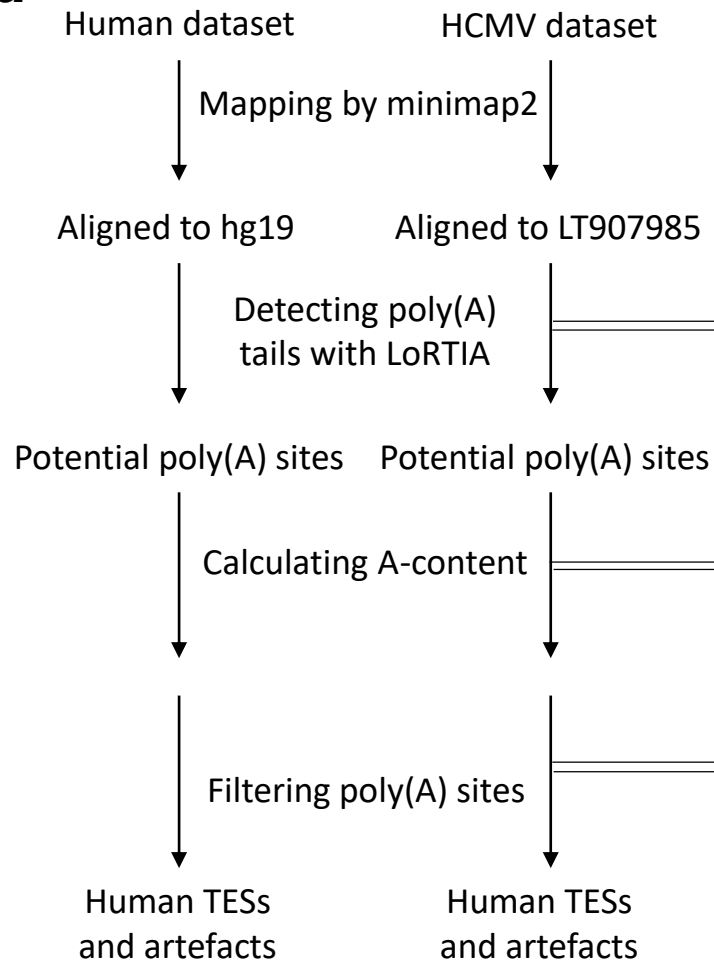**b**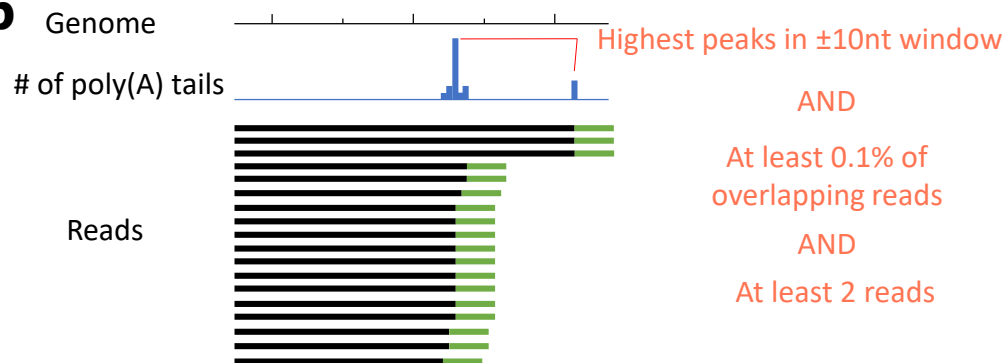**c**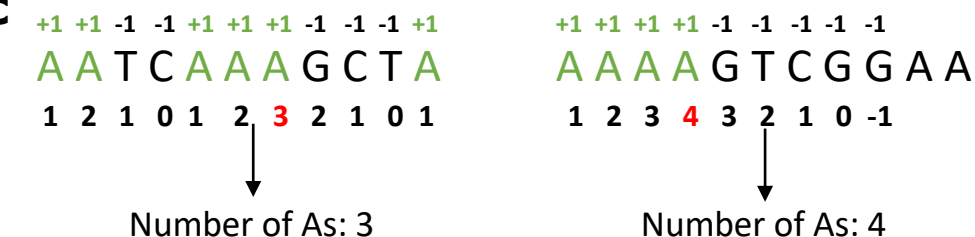**d**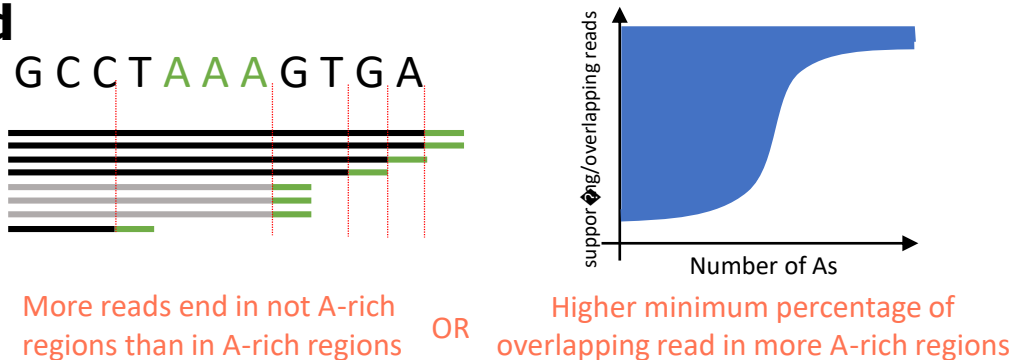

Supplement: Supplementary file 2 — Additional file 2: Figure S2. Overview of the analysis. (a) A summary of the pipeline. Specific steps are explained in detail in boxes. (b) Identifying potential poly(A) sites. The criteria used by the LoRTIA toolkit to detect poly(A) sites are listed. The number of polyadenylated reads ending at a given nucleotide had to be at least two and at least 0.1% of the overlapping reads for the site to be considered a potential poly(A) site. If multiple sites fulfilled these criteria in a 21-nt window, the site with the highest number of supporting reads was accepted. (c) The algorithm for determining the adenine content of a region. The counting started from the poly(A) site, and each A increased, while every different letter decreased the counter by one. The counting continued until maximum 20 nucleotides or until the counter reached − 1. The highest value of the counter was regarded as the adenine content. (d) Criteria for characterizing a potential poly(A) site as a TES. One criterion was that the number of reads ending in not A-rich regions (less than three As) in a 21-nt window, had to be higher than the number of reads ending in A-rich regions (three or more As). The second criterion was that the number of supporting reads had to be higher than the percentage of overlapping reads calculated by a logistic function that takes into consideration the adenine content of the region. A potential poly(A) site was classified as a TES if any of the two criteria was fulfilled. [file 12864_2019_6199_MOESM2_ESM.pdf]
