# Supplementary material for: Template-switching artifacts resemble alternative polyadenylation
Source: BMC Genomics. 2019 Nov 8;20:824. doi: 10.1186/s12864-019-6199-7 (PMC6839120; doi:10.1186/s12864-019-6199-7)
Supplement: Supplementary file 3 — Additional file 3: Table S1. Negative predictive value in A-rich regions in the human dataset. The percentage of putative TS artifacts not supported by the dRNA sequencing experiment (i.e. negative predictive value) is presented in A-rich regions of different adenine content calculated by two algorithms. If artifacts were mainly caused by internal priming, it would be expected that true negative calls accumulated in regions which have a high number of adenines despite having few consecutive adenines close to the artifactual pA site. The cells are colored according to their negative predictive value. [file 12864_2019_6199_MOESM3_ESM.pdf]

Supplementary Table 1. True negative rate in A-rich regions in the human dataset. The percentage of putative TS artifacts not supported by the dRNA sequencing experiment (i.e. true negativity rate) is presented in A-rich regions of different adenine content calculated by two algorithms. If artifacts are were mainly caused by internal priming, it would be expected that true negative calls accumulated in regions which have a high number of adenines despite having few consecutive adenines close to the artifactual pA site. The cells are colored according to their true negativity rate.

Number of adenines (TS algorithm)	Number of adenines in 20 nucleotides																	
	3	4	5	6	7	8	9	10	11	12	13	14	15	16	17	18	19	20
3	86.36%	83.67%	69.05%	73.47%	65.10%	64.92%	59.82%	50.36%	52.05%	43.48%	42.86%	0.00%	-	-	-	-	-	-
4	-	85.71%	90.48%	76.06%	76.65%	64.69%	67.32%	62.92%	63.37%	66.25%	75.00%	100.00%	-	-	-	-	-	-
5	-	-	75.00%	73.08%	79.33%	76.71%	73.93%	76.78%	70.10%	68.16%	100.00%	-	-	-	-	-	-	-
6	-	-	-	85.71%	78.43%	75.86%	81.01%	76.56%	73.46%	73.90%	72.15%	-	-	-	-	-	-	-
7	-	-	-	-	87.50%	78.13%	80.38%	83.62%	81.55%	80.64%	75.33%	-	-	-	-	-	-	-
8	-	-	-	-	-	82.61%	80.60%	82.08%	83.06%	84.98%	84.87%	73.75%	-	-	-	-	-	-
9	-	-	-	-	-	-	78.13%	88.10%	84.58%	89.39%	85.98%	85.71%	-	-	-	-	-	-
10	-	-	-	-	-	-	-	77.36%	85.71%	85.60%	85.79%	83.20%	81.57%	-	-	-	-	-
11	-	-	-	-	-	-	-	-	92.86%	89.04%	85.48%	84.51%	83.81%	-	-	-	-	-
12	-	-	-	-	-	-	-	-	-	92.39%	91.28%	84.82%	86.31%	86.81%	-	-	-	-
13	-	-	-	-	-	-	-	-	-	-	93.71%	90.02%	87.50%	88.80%	-	-	-	-
14	-	-	-	-	-	-	-	-	-	-	-	96.16%	93.30%	89.80%	90.65%	-	-	-
15	-	-	-	-	-	-	-	-	-	-	-	-	94.02%	93.85%	90.14%	-	-	-
16	-	-	-	-	-	-	-	-	-	-	-	-	-	95.96%	93.91%	95.05%	-	-
17	-	-	-	-	-	-	-	-	-	-	-	-	-	-	96.94%	94.80%	-	-
18	-	-	-	-	-	-	-	-	-	-	-	-	-	-	-	98.12%	97.13%	-
19	-	-	-	-	-	-	-	-	-	-	-	-	-	-	-	-	96.91%	-
20	-	-	-	-	-	-	-	-	-	-	-	-	-	-	-	-	-	99.34%
